# BA71ΔCD2: a New Recombinant Live Attenuated African Swine Fever Virus with Cross-Protective Capabilities

**DOI:** 10.1128/JVI.01058-17

**Published:** 2017-10-13

**Authors:** Paula L. Monteagudo, Anna Lacasta, Elisabeth López, Laia Bosch, Javier Collado, Sonia Pina-Pedrero, Florencia Correa-Fiz, Francesc Accensi, María Jesús Navas, Enric Vidal, María J. Bustos, Javier M. Rodríguez, Andreas Gallei, Veljko Nikolin, María L. Salas, Fernando Rodríguez

**Affiliations:** a IRTA, Centre de Recerca en Sanitat Animal (CReSA, IRTA-UAB), Campus de la Universitat Autònoma de Barcelona, Bellaterra, Spain; b UAB, Centre de Recerca en Sanitat Animal (CReSA, IRTA-UAB), Campus de la Universitat Autònoma de Barcelona, Bellaterra, Spain; c Departament de Sanitat i Anatomia Animals, Facultat de Veterinària, UAB, Bellaterra, Barcelona, Spain; d Centro de Biología Molecular Severo Ochoa, Consejo Superior de Investigaciones Científicas and Universidad Autònoma de Madrid, Madrid, Spain; e Centro Nacional de Microbiología (ISCIII), Majadahonda, Madrid, Spain; f Boehringer Ingelheim Veterinary Research Center GmbH & Co. KG, Hannover, Germany; University of Southern California

**Keywords:** African swine fever virus, CD8 T cells, cross-protection, live attenuated virus, vaccine

## Abstract

African swine fever is a highly contagious viral disease of mandatory declaration to the World Organization for Animal Health (OIE). The lack of available vaccines makes its control difficult; thus, African swine fever virus (ASFV) represents a major threat to the swine industry. Inactivated vaccines do not confer solid protection against ASFV. Conversely, live attenuated viruses (LAV), either naturally isolated or obtained by genetic manipulation, have demonstrated reliable protection against homologous ASFV strains, although little or no protection has been demonstrated against heterologous viruses. Safety concerns are a major issue for the use of ASFV attenuated vaccine candidates and have hampered their implementation in the field so far. While trying to develop safer and efficient ASFV vaccines, we found that the deletion of the viral CD2v (EP402R) gene highly attenuated the virulent BA71 strain *in vivo*. Inoculation of pigs with the deletion mutant virus BA71ΔCD2 conferred protection not only against lethal challenge with the parental BA71 but also against the heterologous E75 (both genotype I strains). The protection induced was dose dependent, and the cross-protection observed *in vivo* correlated with the ability of BA71ΔCD2 to induce specific CD8^+^ T cells capable of recognizing both BA71 and E75 viruses *in vitro*. Interestingly, 100% of the pigs immunized with BA71ΔCD2 also survived lethal challenge with Georgia 2007/1, the genotype II strain of ASFV currently circulating in continental Europe. These results open new avenues to design ASFV cross-protective vaccines, essential to fight ASFV in areas where the virus is endemic and where multiple viruses are circulating.

**IMPORTANCE** African swine fever virus (ASFV) remains enzootic in most countries of Sub-Saharan Africa, today representing a major threat for the development of their swine industry. The uncontrolled presence of ASFV has favored its periodic exportation to other countries, the last event being in Georgia in 2007. Since then, ASFV has spread toward neighboring countries, reaching the European Union's east border in 2014. The lack of available vaccines against ASFV makes its control difficult; so far, only live attenuated viruses have demonstrated solid protection against homologous experimental challenges, but they have failed at inducing solid cross-protective immunity against heterologous viruses. Here we describe a new LAV candidate with unique cross-protective abilities: BA71ΔCD2. Inoculation of BA71ΔCD2 protected pigs not only against experimental challenge with BA71, the virulent parental strain, but also against heterologous viruses, including Georgia 2007/1, the genotype II strain of ASFV currently circulating in Eastern Europe.

## INTRODUCTION

African swine fever (ASF) is a devastating and highly contagious disease listed by the World Organization for Animal Health (OIE) as a disease of obligatory declaration, and its causative agent, African swine fever virus (ASFV), has been included as a biothreat agent by the authorities of the United States (1). ASFV has remained enzootic in Africa for decades following a sylvatic cycle between domestic pigs and the ASFV natural reservoirs, African wild pigs and Ornithodoros ticks, in which the virus can replicate without apparent clinical signs (2). The uncontrolled presence of ASFV in Africa has facilitated its periodic exportation to other parts of the world, including the first and second entrances into the Iberian Peninsula in 1957 and 1968, where it became enzootic for almost 40 years. In 2007, only 10 years after its eradication from the European continent (ASFV remains enzootic in Sardinia), ASFV arrived to the Caucasian Republic of Georgia from East Africa, again as a food contaminant (3). Since then, ASFV has spread across the Caucasian Region and the Russian Federation (4, 5); more recently, several outbreaks have been declared in some Eastern European Union countries (6, 7), mainly associated with wild boar movements (8, 9). The lack of available vaccines against ASFV limits the ASFV control to good management, early diagnosis, and, in the case of wealthy ASF-free areas, the massive culling of infected and in-contact pigs, strategies that are being continuously reviewed (10, 11). Independent of ethical considerations, stamping-out policies are out of reach for less favored countries where ASFV is enzootic, including most areas of Sub-Saharan Africa and Eastern Europe, thus complicating even more the control of a disease that is contributing to poverty and swine industry underdevelopment (12, 13). Obtaining a safe and efficacious vaccine against ASFV would help to reduce the ASFV pressure in areas where it is enzootic, alleviate their economy, and reduce the chances of further exportation of the virus to ASF-free countries, thus positively affecting global commercialization exchanges. The complexity of ASFV, a large enveloped double-stranded DNA virus that encodes more than 150 different proteins, and ASFV tropism for the pig macrophage (14), a key cellular component of the immune system, have complicated this task.

Work performed decades ago clearly demonstrated that experimental vaccines based on physical or chemical inactivated ASFV failed to induce solid protective immunity (15–17), an observation that has been confirmed using more modern adjuvants (18). Conversely, live attenuated ASFV isolates, either naturally isolated or obtained by tissue culture adaptation, have been demonstrated to confer solid protection against experimental infections with homologous virulent viruses (19, 20) and only in a limited manner against heterologous ASFV challenges (21–23). Safety concerns have thus far hampered their field implementation, but live attenuated viruses (LAVs) have become essential tools to better understand the role that both specific antibodies and T cells play in ASF protection. On the one hand, passive transfer of anti-ASFV IgG from ASFV-surviving pigs allowed naive pigs to survive homologous ASFV challenge (24). On the other hand, ASFV-surviving pigs immune depleted of their CD8^+^ T cells did not resist lethal homologous ASFV challenge (25–27). Other T-cell subsets, including innate and adaptive T-regulatory cells and other arms of the innate immune system, including NK cells, also seem to play an important role in protection (28, 29). The most recent incorporation of molecular technologies has allowed the precise manipulation of the ASFV genome by homologous recombination, thus obtaining ASFV deletion mutants lacking nonessential genes (30) and, more recently, recombinant viruses encoding essential ASFV genes under the control of inducible promoters (31). These methodologies have allowed a better understanding of key aspects of ASFV biology, including ASFV morphogenesis (32), and have opened new avenues for rational vaccine development. The European Commission has recently confirmed the use of attenuated strains as the most plausible approach to develop an effective ASF vaccine in the short/medium term (https://ec.europa.eu/food/animals/animal-diseases/control-measures_en).

The presence of multiple ASFV genes involved in host immune evasion (29, 33) confirms the relevance of the innate immune system in protection and has allowed the design of live attenuated ASFVs with vaccine potential by specifically deleting one or more of these virulence factors from different virulent ASFV strains, including Georgia 2007/1 (34–36). All these recombinant vaccine prototypes have demonstrated solid protection against experimental challenge with the parental virulent viruses (homologous challenges), but again, safety is their major concern. A much safer vaccine alternative to LAVs could be the use of single-cycle and replication-competent ASFV, as developed for other viruses. Original work performed in our laboratory has allowed, so far, the generation of recombinant viruses containing ASFV essential genes under the control of an inducible promoter, by replacing, first, the nonessential thymidine kinase (TK) gene with the repressor cassette under the control of an ASFV promoter (31). Thanks to this technique, we have learned the role that several structural proteins play during ASFV morphogenesis in infected Vero cells, including the polyprotein pp220 (37). BA71VΔTKv220i, the inducible BA71V virus (Vero cell-adapted virus strain), expresses the essential structural pp220 polyprotein only when the isopropyl-β-d-thiogalactopyranoside (IPTG) inducer is present. In the absence of IPTG (as would happen *in vivo*), the infected Vero cells produced DNA-empty capsids that can be purified from the extracellular milieu (37). As mentioned above, this single-cycle infection strategy could theoretically reduce to the minimum the safety problems inherited by the infectious nature of LAVs.

Adaptation of ASFV to Vero cells has been very useful to understand many aspects of its cell cycle and to develop gene manipulation strategies that allowed unmasking the function of many ASFV proteins *in vitro* but yields nonpathogenic viruses after serial passages, as demonstrated for BA71 and Georgia 2007/1 (38). Therefore, the innocuous nature of the nonpathogenic BA71V also prevents the use of available BA71V-based recombinant viruses (30–32) as potential vaccines, including the inducible virus BA71VΔTKv220i (37).

By following the same methodology as used for BA71V, in this study, we have obtained a new virus inducible for the pp220 protein using the BA71 field strain and a stable COS-1 cell line. The fact that BA71 needs no adaptation period to grow in COS-1 cells (39) significantly increases its potential use for vaccine purposes.

While trying to characterize the protective potential of inducible ASF viruses, we confirmed in this study that the TK gene is essential for BA71 replication in macrophages and that those inducible viruses deficient in TK induce inefficient specific immune responses and afford no protection against homologous BA71 challenge. Searching for an alternative nonessential locus to generate future inducible viruses, we also found a new live attenuated ASFV with interesting cross-protective capabilities.

Thus, we demonstrated the following: first, that deletion of the EP402R open reading frame (ORF) encoding the ASFV hemagglutinin, also known as viral CD2 (CD2v) (40, 41), totally attenuated the virulent BA71 ASFV strain; second, that the deletion mutant BA71ΔCD2 was capable of protecting pigs in a dose-dependent manner, not only against the homologous BA71 virus but also against the heterologous E75 virulent challenge; third, that the protection afforded correlated with the ability of BA71ΔCD2 to induce CD8^+^ T cells that specifically recognized both viruses *in vitro*; and fourth, that BA71ΔCD2 also protected against the ASFV challenge with Georgia 2007/1, the virus currently circulating in Eastern Europe and belonging to genotype II (BA71 and E75 belong to genotype I). The fact that BA71 and BA71ΔCD2 could grow equally well in the COS-1 cell line and in porcine alveolar macrophages (PAMs) without any effect on their genetic stability, pathogenicity, or immunogenicity is of great interest in terms of large-scale manufacturing production. In spite of these promising results, there is still room for improvement, mainly from the biosafety point of view.

## RESULTS

### BA71 grown in COS-1 cells remains infectious *in vivo*.

The use of primary porcine macrophages is not an attractive option for industrial partners, so the first experiment performed was aimed at confirming that BA71 grown in COS-1 cells remained infectious *in vivo*. No significant differences were observed *in vivo* after inoculation of pigs with different doses of BA71, grown either in COS-1 cells (BA71-Cos) or in PAMs (BA71). All pigs died within 2 weeks of the inoculation, even after receiving a single dose of 100 PFU (Table 1), 10^5^-fold lower than the dosage used for BA71V, used as a control (Table 1). As expected, large doses of the nonpathogenic BA71V were incapable of infecting pigs or inducing specific immune responses, thus confirming the innocuous nature of this virus.

**TABLE 1:** BA71 grown in COS-1 cells remains infectious *in vivo*^*a*^

Virus	Cells used for *in vitro* amplification	No. of intramuscular doses (dose, PFU)	Clinical outcome (mortality rate, %)	ASFV-specific immune response	% protection after BA71 lethal challenge
BA71	PAMs	1 (100)	Acute ASF (100)	−/+	NP
BA71V	Vero cells	2 (10^7^)	Asymptomatic (0)	None	0
BA71-Cos	COS-1 cells	1 (100)	Acute ASFV (100)	−/+	NP
BA71ΔTKv220i	COS-1 cells	2 (10^7^)	Asymptomatic (0)	None	0
BA71ΔTK	COS-1 cells	2 (10^7^)	Asymptomatic (0)	None	0

a Groups of 6 animals were inoculated with different quantities and doses of the corresponding recombinant viruses and next challenged with a lethal dose of BA71. ASF clinical signs (including virus detection) and mortality were recorded. −/+, low but detectable anti-ASFV antibodies at time of death; pigs initially inoculated with BA71 and BA71-Cos died of acute ASFV, so rechallenge with BA71 could not be performed (NP).

Once the feasibility of BA71 growth in COS-1 cells was confirmed without significantly losing its virulence, we generated the inducible BA71ΔTKv220i, a recombinant BA71 virus encoding the pp220 viral polyprotein under the control of an IPTG-inducible promoter, on COS-1 cells. Pigs inoculated twice with either 10^5^ or 10^7^ PFU of BA71ΔTKv220i did not develop any clinical signs compatible with ASF, and no recombinant virus was detectable at any time postinfection (p.i.) in any of the evaluated samples, thus confirming its safety (Table 1). Unexpectedly, all pigs immunized with BA71ΔTKv220i died suddenly or had to be sacrificed for humanitarian reasons before day 7 after lethal BA71 challenge, showing clinical signs similar to those in control nonimmunized animals (Table 1). Correlating with the lack of protection afforded, pigs inoculated with 10^7^ PFU BA71ΔTKv220, failed to show specific immune responses (Table 1). Additional *in vivo* experiments performed administering BA71ΔTK, the TK deletion mutant classically used as an intermediary virus to generate inducible viruses (31), allowed the demonstration of the incapability of this virus to replicate and/or to induce ASFV-specific humoral or cellular responses *in vivo* (Table 1). In conclusion, it is necessary to find an alternative nonessential locus within the ASFV genome for the future generation of inducible viruses for vaccine purposes.

### TK, but not CD2v, is essential for BA71 macrophage infection.

**FIG 1:**
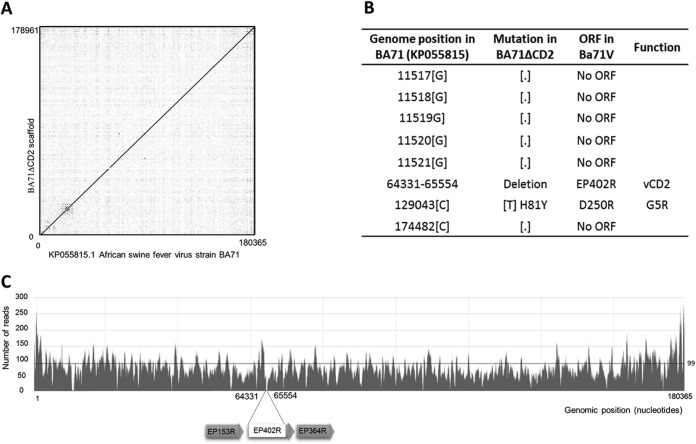
BA71ΔCD2 is genetically stable in COS-1 cells. (A) Dot plot representing the conservation of the sequence of BA71ΔCD2 after passages in COS-1 cells compared with the BA71 parental strain. The continuous line of dots with a slope of 1 represents the undisturbed segment of conservation, disrupted only in the region where the EP402R gene was deleted. (B) Detailed table showing the differences observed between the genomes. (C) Depth of the genome sequence obtained. The partial deletion of the EP402R gene and the two genes flanking the region are schematically represented below the plot.

At the time this work was performed, few viral genes had been described as nonessential for ASFV *in vivo* replication. The first ASFV deletion mutant successfully tested *in vivo* was delta8-DR, a recombinant virus lacking the hemagglutinin, also known as CD2v (40, 41), that is based on the Malawi Lil-20/1 isolate and that showed reduced viremia but clinical signs and mortality similar to those of the parental virus (42). With these data at hand, a new deletion mutant virus, BA71ΔCD2, was obtained in COS-1 cells using the BA71 virulent virus, following protocols described in Materials and Methods. Therefore, the BA71ΔCD2 recombinant virus has the EP402 ORF replaced with the Lac repressor cassette and will serve as an intermediary construct for the future generation of inducible viruses. Aiming to confirm the integrity and stability of the BA71ΔCD2 genome in COS-1 cells, a virus stock was sequenced after 20 passages in COS-1 cells. A single genome contig was obtained after trimming, mapping, and assembling/scaffolding of the reads obtained through massive sequencing. As shown in Fig. 1A, no significant changes were found in the BA71ΔCD2 genome, with the exception of those artificially introduced by genetic recombination and seven point mutations with only one mapping in a known ORF, D250R, and encoding a decapping enzyme (43) with histidine 81 changed to a tyrosine (Fig. 1B). The median depth of the sequence obtained was 99×, although a larger number of reads were detected in both left and right ends, corresponding to the presence of large repetitive sequences within the ASFV genome (Fig. 1C). All the experiments described below were performed with this virus stock.

**FIG 2:**
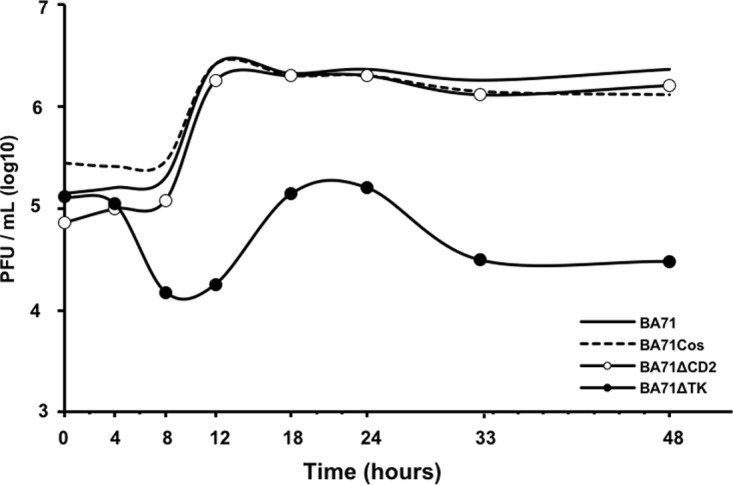
TK is essential for BA71 growing in porcine alveolar macrophages (PAMs) *in vitro*, while CD2v is not. PAMs were infected at a multiplicity of infection (MOI) of 1 with the following virus stocks: BA71 (BA71 grown in macrophages), BA71-Cos (BA71 grown in COS-1 cells), BA71ΔCD2 (BA71Cos deficient in the CD2v gene grown in COS-1 cells), and BA71ΔTK (BA71-Cos lacking the thymidine kinase gene and grown in COS-1 cells), and the kinetics of virus production was moniotored by harvesting of the cell supernatants at different times. All samples were directly titrated by plaque assay in COS-1 cells, and the results were plotted in the logarithmic scale as PFU per milliliter of cell supernatant.

BA71ΔCD2 showed growth kinetic curves in PAMs similar to those of the parental BA71 virus, grown either in COS-1 cells or in PAMs (Fig. 2). Compared with the exponential growth curves observed for BA71ΔCD2, little, if any, virus was produced by BA71ΔTK-infected macrophages (Fig. 2). Thus, BA71ΔCD2 was tested for its replication competence and virulence *in vivo*.

### CD2v deletion attenuates BA71 *in vivo*.

**FIG 3:**
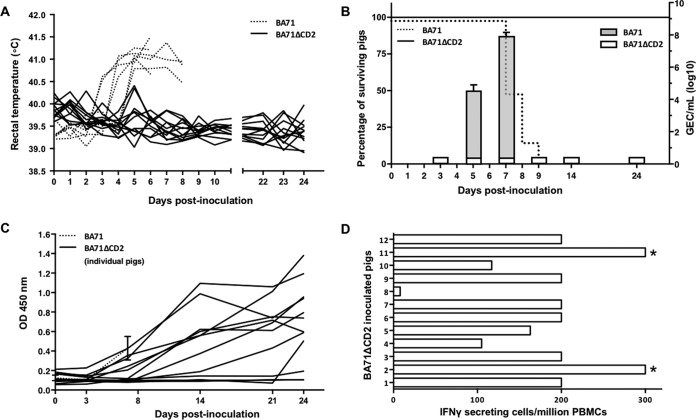
CD2v deletion attenuates BA71. Pigs were immunized with 10^3^ PFU of either BA71 (discontinuous lines) or BA71ΔCD2 (continuous lines) and different parameters were monitored, including fever, viremia, death, and induction of specific antibodies and T cells. (A) Rectal temperatures were taken for individual pigs at different days after infection with either BA71 (discontinuous line) or BA71ΔCD2 (continuous line). (B) Average viremia kinetics from pigs immunized with BA71 (gray bars) or BA71ΔCD2 (white bars) are represented together with their corresponding standard deviations (right *y* axis). Virus titers are represented in the logarithmic scale as genome-equivalent copies per milliliter of serum. The percentage of surviving pigs after infection with BA71 (discontinuous line) or BA71ΔCD2 (continuous line) is also represented (left *y* axis). (C) The induction of ASFV-specific antibodies was measured by ELISA, and data corresponding to the kinetics of antibody induction for the 12 individual pigs inoculated with BA71ΔCD2 (continuous lines) are shown as optical density values. Averages and standard deviations for 6 pigs infected with BA71 are also represented. (D) Data correspond to the number of ASFV-specific T cells detectable in blood of the 12 pigs inoculated with BA71ΔCD2 24 days after its administration. Values correspond to the number of T cells that specifically secrete IFN-γ after BA71ΔCD2 *in vitro* stimulation per million peripheral blood mononuclear cells (PBMCs). *, ≥300 IFN-γ-secreting cells.

To evaluate the *in vivo* effect of the deletion of CD2 from the BA71 virulent strain, two groups of pigs were inoculated with either 10^3^ PFU of BA71 (20 median lethal doses [LD_50_]), the dose of virus normally used in our lethal challenge experiments, or 10^3^ PFU of BA71ΔCD2. As expected, animals infected with the parental BA71 virus exhibited clinical signs associated with acute ASF, including fever (Fig. 3A) and high viremia detectable in serum by quantitative PCR (qPCR) from day 4 p.i., which increased progressively over time until the animals died or were euthanized before day 9 p.i. (Fig. 3B). In clear contrast, and converse to results shown for delta8-DR from the Malawi strain (42), pigs inoculated with the same dose of BA71ΔCD2 remained alive until the end of the experiment (24 days), showing no clinical signs compatible with ASF and no detectable viremia during the entire period of observation (Fig. 3A and B).

Inoculation with as low as 10^3^ PFU of BA71ΔCD2 induced specific antibodies that were detectable from day 7 p.i., showing at this time levels similar to those found in moribund pigs inoculated with 10^3^ PFU of BA71 (Fig. 3C). Interestingly, BA71ΔCD2-immunized pigs showed increasing titers of ASFV-specific antibodies; titers reached their maximum by day 24 p.i. in 10 out of 12 inoculated pigs (Fig. 3C). Similarly, a significant number of ASFV-specific T cells was detectable in 11 out of 12 pigs by day 24 p.i. (Fig. 3D), while no specific T-cell responses were detectable in control pigs infected with BA71 (data not shown). The fast and efficient seroconversion and the induction of specific T cells observed after inoculation with 10^3^ PFU of BA71ΔCD2 contrast strongly with the lack of specific immune responses observed after two *in vivo* inoculations with 3- to 4-log-higher doses of either BA71V, BA71ΔTKv220i, or BA71ΔTK (Table 1) and demonstrate that BA71ΔCD2 is attenuated and induces a strong humoral and cellular immune response against ASFV *in vivo*.

### BA71ΔCD2 protects in a dose-dependent manner against BA71 homologous lethal challenge.

**FIG 4:**
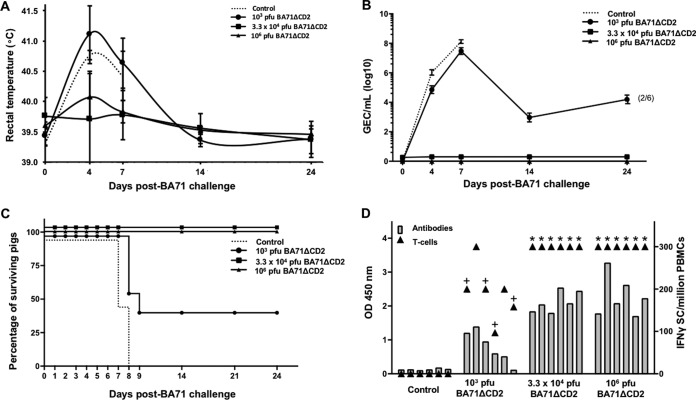
Homologous protection afforded by BA71ΔCD2. The degree of protection afforded by the different doses of BA71ΔCD2 (continuous lines) after BA71 lethal challenge is shown by comparing the fever (A) and the viremia (B) kinetics after BA71 challenge and monitoring the proportion of surviving pigs left in each group (C). Data plotted in panels A and B correspond to average values per animal group and time tested, while error bars represent the standard deviations. The levels of ASFV-specific antibodies (gray bars, left *y* axis) and T cells (▲, right *y* axis) found in the blood of the individual pigs just before BA71 challenge are also shown (D). Control, challenge control (discontinuous lines). *, ≥300 IFN-γ-secreting cells; *+*, BA71ΔCD2-inoculated pigs not protected against BA71 lethal challenge.

Aiming to characterize the protective potential of BA71ΔCD2, groups of 6 pigs were inoculated with either 10^3^, 3.3 × 10^4^, or 10^6^ PFU of the CD2v-deficient virus. A group of 6 nonimmunized pigs was also included, and all animals were challenged with a lethal dose of 10^3^ hemagglutinating units (HAU) of the homologous virulent BA71 virus 24 days later. As expected, control pigs infected with BA71 developed severe ASF clinical signs, evident from day 3 to 5 p.i., including fever (Fig. 4A) and high viremia (Fig. 4B), and all animals died before day 8 p.i. (Fig. 4C). In clear contrast, pigs inoculated with the intermediate or high doses of BA71ΔCD2 did not show significant ASF clinical signs or viremia at any time postinfection, thus demonstrating the solid protection afforded. Inoculation of 10^3^ PFU of BA71ΔCD2 showed partial protection in two out of six vaccinated animals (33%) surviving the homologous lethal challenge, and one of them showed no clinical signs or viremia at any time postinfection.

With the exception of one animal (pig number 5, inoculated with 10^3^ PFU of BA71ΔCD2) that survived despite the absence of detectable antibodies at the time of BA71 challenge, the protection afforded by BA71ΔCD2, correlated with the presence of specific humoral and cellular responses (Fig. 4D) before BA71 challenge. Moreover, all pigs surviving the challenge showed detectable immune responses at the time of sacrifice (data not shown).

### BA71ΔCD2 confers protection against heterologous E75 lethal challenge, correlating with the induction of cross-reactive CD8^+^ T cells.

**FIG 5:**
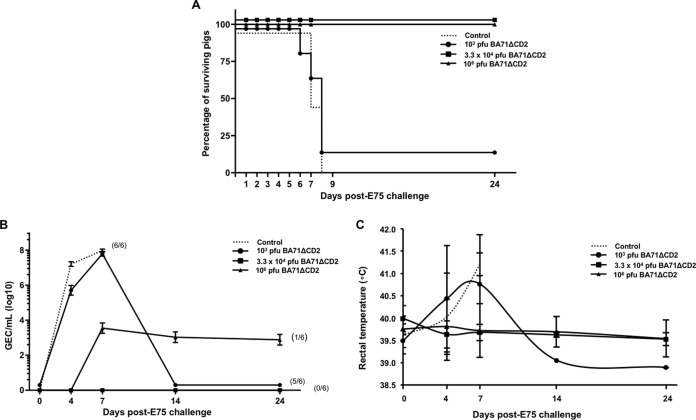
Heterologous protection afforded by BA71ΔCD2. The degrees of protection afforded by the different doses of BA71ΔCD2 after E75 lethal challenge were evaluated by moniotoring the proportion of surviving pigs left in each group (A) and comparing the viremia (B) and the fever (C) kinetics after E75 challenge. Data plotted in panels B and C correspond to average values per animal group and time tested, while error bars represent the standard deviations. Control, challenge control (discontinuous lines) and groups with different doses of BA71ΔCD2 (continuous lines).

With the aim of characterizing the cross-protective potential of BA71ΔCD2, three groups of 6 pigs each were inoculated with different doses of the CD2v-deficient virus—10^3^, 3.3 × 10^4^, or 10^6^ PFU—and were challenged with a lethal dose of 10^4^ HAU of E75 24 days later. In contrast to the lack of protection afforded against the heterologous BA71 ASFV strain by the previously used E75CV1 attenuated virus (20), immunization with either 3.3 × 10^4^ or 10^6^ PFU of BA71ΔCD2 fully protected all 12 pigs against heterologous E75 lethal challenge, with 100% of the immunized pigs surviving (Fig. 5A). Interestingly, all 6 pigs inoculated with the intermediate dose of 3.3 × 10^4^ PFU remained free of clinical signs compatible with ASF after E75 challenge and showed no viremia throughout the infection, while 1 out of 6 pigs receiving the maximum dose showed a peak of mild fever as a unique clinical sign, coinciding with the detection of low viremia from day 7 to 24 p.i., about 4 logs lower than in the control pigs (Fig. 5B). Similar to the experiment described above, 10^3^ PFU of BA71ΔCD2 conferred partial protection, with only one survivor (out of six immunized pigs) after the heterologous E75 challenge (Fig. 5).

**FIG 6:**
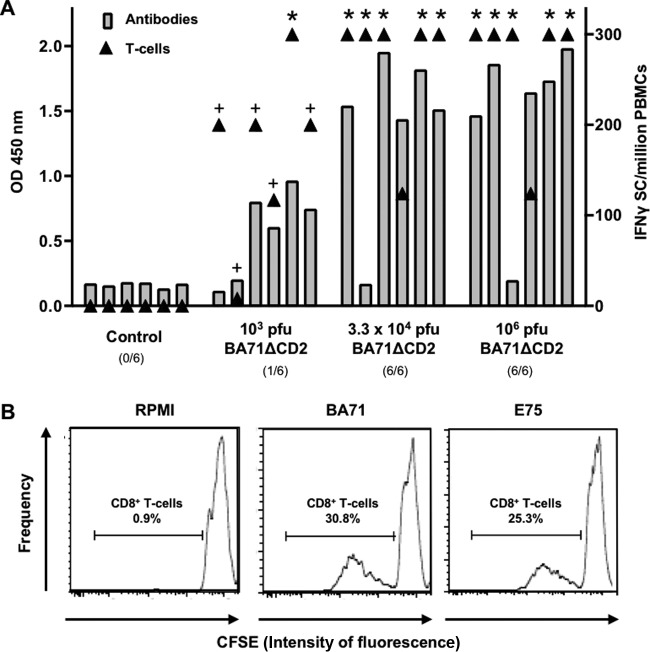
Cross-protection correlates with the ability of BA71ΔCD2 to induce cross-reactive CD8^+^ T cells. The levels of ASFV-specific antibodies (gray bars, left *y* axis) and T cells (▲, right *y* axis) found in the blood of individual BA71ΔCD2-inoculated pigs just before E75 challenge are shown (A). The percentage of CD8^+^ T cells specifically proliferating in response to *in vitro* BA71 or E75 stimulation is also shown (B). The histogram displays the results obtained from a BA71ΔCD2-inoculated pig that survived E75 challenge, using PBMCs obtained just before E75 challenge. *, ≥300 IFN-γ secreting cells; +, BA71ΔCD2-inoculated pigs not protected against E75 lethal challenge.

The detection of high levels of ASFV-specific immune responses after BA71ΔCD2 vaccination has a relative value as a protection predictor when evaluated at a group level, but once more, a lack of total correlation was observed between protection and the level of ASFV-specific immunity present at the time of E75 challenge, detectable by enzyme-linked immunosorbent assay (ELISA) and gamma interferon (IFN-γ) enzyme-linked immunosorbent spot (ELISPOT) assay (Fig. 6A). Thus, some pigs inoculated with low doses of BA71ΔCD2 succumbed to the ASFV challenge in spite of the presence of a high number of specific T cells. Similarly, two surviving pigs, one immunized with the intermediate and the other with the high dose of BA71ΔCD2, showed nondetectable specific antibodies at the time of challenge (Fig. 5A). Aiming to further explore the mechanisms behind the observed cross-protection against E75, a 5-day *in vitro* carboxyfluorescein diacetate succinimidyl ester (CFSE) proliferation assay was performed using peripheral blood mononuclear cells (PBMCs) obtained at day 24 post-BA71ΔCD2 inoculation (just before E75 challenge), using BA71 or E75 as a specific stimulus. Interestingly, every single pig inoculated with BA71ΔCD2 showed specific CD8^+^ T cells in their blood, capable of proliferating *in vitro* in response to either BA71 or E75 (Fig. 6B), although their proportion was extremely variable depending on the animal and the stimulus used (9 to 39%). The ability of BA71ΔCD2 to induce cross-reactive CD8^+^ T cells contrasts with the E75-restricted repertoire induced by the classically attenuated E75CV1 virus (20) and correlates with the differential protective capabilities of both LAVs: BA71ΔCD2 protected against both BA71 and E75 lethal challenges, while E75CV1 exclusively protected against the parental E75 virulent virus but did not protect against BA71 (20).

### BA71ΔCD2 immunization protects against Georgia 2007/1 challenge.

**FIG 7:**
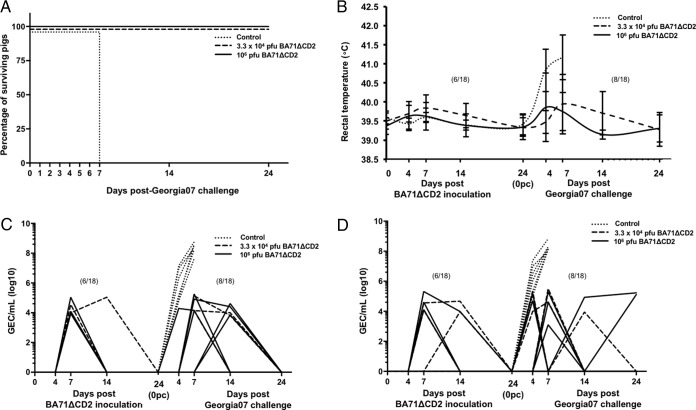
BA71ΔCD2 protects against Georgia 2007/1 heterologous challenge. The degrees of protection afforded by the different doses of BA71ΔCD2 were evaluated after Georgia 2007/1 lethal challenge by monitoring the proportion of surviving pigs left in each group (A) and by comparing the kinetics of fever (B), viremia (C), and ASFV nasal secretion (D). Data plotted in panels B, C, and D correspond to individual animals, and fever, viremia, and nasal secretion were monitored not only after Georgia 2007/1 challenge but also from the day of BA71ΔCD2 immunization. The proportion of pigs with fever and/or ASFV positive, before and/or after challenge, is shown in parentheses. Control, challenge control (discontinuous lines).

Interestingly, when pigs were inoculated with either 3.3 × 10^4^ or 10^6^ PFU of BA71ΔCD2, 100% survived a lethal Georgia 2007/1 challenge (Fig. 7A). Confirming this protective efficacy, 10 out of the 18 pigs receiving BA71ΔCD2 did not show any ASF clinical sign, and no viremia was detectable, while the remaining 8 showed reduced peaks of viremia (Fig. 7C) and nasal shedding (Fig. 7D), reaching maximum virus titers 4 to 5 logarithms lower than those found for control pigs. As expected, virus presence matched well with fever appearance (Fig. 7B). No other ASF-compatible clinical sign was recorded for surviving animals.

Despite the impressive protection afforded, BA71ΔCD2 retains limited but detectable residual virulence. Thus, one-third of the pigs immunized with either 3.3 × 10^4^ or 10^6^ PFU of BA71ΔCD2 did show a very limited but detectable presence of the LAV in blood (Fig. 6C) and in nasal secretions (Fig. 6D). With the exception of brief episodes of fever that coincided with the presence of virus in physiological fluids (Fig. 6C), no other clinical signs compatible with ASF were observed at any time after BA71ΔCD2 inoculation, and all pigs were free of BA71ΔCD2 at the time of Georgia 2007/1 lethal challenge.

## DISCUSSION

The cross-protective capability shown by BA71ΔCD2 allows, at least partially, the circumvention of this major limitation of available ASF experimental vaccine prototypes. The genetic stability observed for BA71ΔCD2 after 20 consecutive passages in COS-1 cells allows us to hypothesize about the future standardization of the large-scale production of this vaccine prototype. Preliminary results obtained in the laboratory seemed to confirm this hypothesis, since supernatants directly obtained from macrophages and COS-1 cells infected with BA71ΔCD2 provided undistinguishable immunogenicity and protective results. ASFV adaptation to Vero and monkey kidney (MS) cells provokes large deletions and mutations in its genome (38, 44–46), yielding nonpathogenic viruses (38, 46). Converse to the resistance of Vero cells or MS cells to become infected by BA71, COS-1 cells are efficiently infected by BA71 (39), and by BA71ΔCD2 without further adaptation, most probably explaining both the genetic (Fig. 1) and functional (Table 1) stability of BA71ΔCD2 *in vivo*.

Obtaining cross-protective vaccines against ASF is desirable for effective ASF control, especially considering areas where ASFV is enzootic, where more than one ASFV isolate could be circulating. The best example of this complex epidemiological picture comes from Africa (47), where 23 ASFV genotypes and a much larger number of ASFV variants have been described so far (48), on occasions concomitantly circulating and sometimes from different genotypes (49, 50). The complex epidemiological situation in the area and the lack of incentives for the farmers make ASF eradication even more difficult and contribute to Africa's poverty (51). With only two ASFV introductions from Africa, in 1957 and 1960, almost 4 decades of ASF being enzootic in the Iberian Peninsula has yielded the appearance of viruses with different degrees of virulence, many of them classified as heterologous due to the lack of cross-protection experimentally observed between them (21). The lack of correlation between cross-protection and ASFV genotyping becomes evident with the absence cross-protection observed for viruses not only belonging to the same genotype but also isolated in the same geographical area (20, 21) and contributes to the complexity of defining ASFV strains as homologous or heterologous. Work performed in the 1980s demonstrated that sera from ASFV-infected convalescent pigs were capable of inhibiting infection by homologous but not heterologous ASFV strains, and as opposed to what was previously believed, the infection inhibition ability did not correspond with the virus-neutralizing capability but rather correlated with the presence of hemagglutination and hemadsorbing inhibitory antibodies (52). Confirming these results, the protective role of the ASFV hemagglutinin (CD2v) and its correlation with the presence of hemagglutination inhibitory antibodies was further confirmed (53, 54), and CD2v together with the c-type lectin antigen has been very recently postulated to be used for serogroup classification of ASFV strains (55). However, together with anti-CD2v/lectin antibodies, other mechanisms might be involved in cross-protection, since viruses with identical CD2v and C-lectin genes have been demonstrated to be heterologous (no cross-protection between them), including the BA71 and E75 Spanish isolates (20). The protection afforded by BA71ΔCD2 or by other nonhemadsorbing ASFV isolates (19) confirms this hypothesis, since no hemagglutination or hemadsorbing inhibitory antibodies could be induced, but cross-protection was observed. The strong correlation observed between BA71ΔCD2-mediated protection and the presence of *in vitro* cross-reactive CD8^+^ T cells (Fig. 6B) adds new light to this paradigm. Depletion studies using anti-CD8 antibodies clearly demonstrate a protective role for cells expressing the CD8 molecule on their surface (25), including different subsets of CD8^+^ T cells but also other cells from the immune system, such as NK cells (56), confirmed by the fact that protection has been afforded even in the absence of specific antibodies (26, 27). The ability of BA71ΔCD2 to induce cross-reactive CD8^+^ T cells contrasts with the E75-restricted CD8^+^ T-cell repertoire induced by the classically attenuated E75CV1 virus (20) and correlates with the differential protective capabilities of both LAVs: BA71ΔCD2 protects against both BA71 and E75 lethal challenges, while E75CV1 exclusively protected against the parental E75 virulent virus but did not protect against BA71, thus defining it as heterologous (20).

Several immunodominant ASFV antigens have already been described as constantly recognized by sera from pigs surviving ASFV infection (57–61); the immunodominant cytotoxic T-lymphocyte (CTL) determinants have so far remained elusive, probably due to the strong immunodominance established in surviving pigs after an ASFV infection (28). The already-described ability of CD2v to inhibit mitogen-dependent lymphocyte proliferation (41) might contribute to the immunodominance observed, thus explaining the ability of BA71ΔCD2 to induce broader and cross-reactive T-cell responses. We are currently extending these studies trying to understand the intrinsic mechanisms explaining the BA71 CD2-immunosupressive abilities. One of the most intriguing questions that still remain unanswered is why the CD2v deletion mutants based on BA71 (BA71ΔCD2) or on Malawi (delta8-DR) strains yielded two recombinant viruses with very different degrees of attenuation. While pigs inoculated with delta8-DR died following kinetics similar to those infected with the Malawi parental strain, doses up to 10^6^ HAU of BA71ΔCD2 yielded almost innocuous results, with only one-third of the immunized animals showing brief fever peaks, the only apparent clinical sign of disease.

The absence of functional CD2v in both BA71ΔCD2 (CD2v ORF totally removed with the exception of 36 bp at the portion corresponding to the carboxyl terminus) and delta8-DR (CD2v ORF totally removed with the exception of 9 bp at the portion corresponding to the amino terminus and 30 bp of 3′ flanking sequence) paves the way for speculation. Differences in the CD2v sequence between Malawi and BA71 might define different functions during ASFV pathogenesis, perhaps with CD2v becoming a more relevant and essential virulence factor for BA71 *in vivo* pathology than for Malawi, in which case its loss would yield a greater effect. In this regard, we would like to bring attention to the 10 repetitions of the domain (PPPKPC) found in the carboxyl terminus of BA71-CD2v, compared with the only 5 found in Malawi; this polypeptide domain has already been described as essential for binding the SH3P7 actin-binding adaptor protein (62), important for T-cell proliferation, cytokine production, and immune responses (63). As suggested before for CD2v with only three PPPKPC repeats (62), duplication of this domain might contribute to a much stronger binding and kidnapping of its substrates. Alternatively, genetic differences between Malawi and BA71, other than those affecting CD2v, might also explain the different pathogenicities of the CD2v knockout (KO) viruses. In this regard, MGF110 deserves further attention, with the genome of the virulent BA71 strain having, again, a lower number of MGF110 gene members (MGF110L-8L, -11L, -12L, -13L, and 14L) than Malawi (46). Additionally, two genes from the MGF360 family (MGF360-4L and -6L) and one from the MGF505 family (MGF505-1R) are absent from the BA71 genome. Within this region, BA71 also lacks X69R, an ORF encoding a small protein of unknown function. The description of several members of these multigene families as virulence and/or host-range determinants complicates the prediction of a unique responsible gene to explain the differences observed *in vivo* for BA71ΔCD2 and delta8-DR. Differences in the terminal inverted regions found in both the left and right variable regions should not be ruled out.

Interestingly, an effect similar to that observed for the CD2v KO mutants has been reported before for the NL gene (64). Deletion of the NL gene strongly attenuated the E70 isolate but failed to provoke any *in vivo* effect in Malawi or Pretoria viruses (65). The sequence similarity between E70 and BA71 Spanish isolates suggests that the same locus might potentially be capable of redundantly covering the lack of CD2v functions in the Malawi delta8-DR strain. In an attempt to better understand the mechanisms of BA71ΔCD2 attenuation, we are currently trying to generate new rescue viral mutants, including ones partially or completely restoring CD2v and the point mutation found in the D250R gene. Independent of the mechanisms behind its attenuation, we believe that the unique cross-protective capability of BA71ΔCD2 deserves further exploration.

Despite the unexpected and strong cross-protection afforded by BA71ΔCD2, there is still room for improvement, mainly from the biosafety point of view and regarding its ability to induce an immune response distinguishable from those detectable in infected animals (differentiating infected from vaccinated animals [DIVA]). BA71ΔCD2 does not induce antibodies against CD2v, thus making it easy to differentiate vaccinated from infected pigs by using a hemadsorption or a hemagglutination inhibition assay. The natural presence of nonhemadsorbing ASFV strains in certain regions of the world and the possibility of occurrence in others require the search for more DIVA markers in the future, besides the presence of antibodies against the positive marker β-glucuronidase (unpublished observation). The success of BA71ΔCD2 relies on its infectious nature, and as described for wild-type ASFV strains, inactivation procedures abolish its protective capabilities (unpublished data). Despite the low degree of adverse effects observed after inoculation with up to 10^6^ PFU of BA71ΔCD2, long-term *in vivo* experiments with larger groups of pigs will be required to ensure its safety. We believe that obtaining efficient and safe LAVs against ASF is closer than ever. ASF research requires continued public and private investment to allow the obtention of LAVs capable of contributing to control of ASFV in regions where it is enzootic.

## MATERIALS AND METHODS

### Cells and viruses.

The established COS-1 and Vero cell lines (ATCC), both isolated from African green monkey kidneys, were cultured at 37°C in Dulbecco's modified Eagle medium (DMEM) supplemented with 5% heat-inactivated fetal calf serum (FCS; Invitrogen), 100 IU of gentamicin/ml (Sigma-Aldrich), 2 mM l-glutamine (Invitrogen), and nonessential amino acids (Invitrogen). Porcine alveolar macrophages (PAMs) were obtained by lung lavage with phosphate-buffered saline (PBS) from healthy pigs and maintained in RPMI 1640 medium (Gibco) supplemented with 10% heat-inactivated FCS (Invitrogen), 100 IU of penicillin/ml (Invitrogen), 100 μg of streptomycin/ml (Invitrogen), and 2 mM l-glutamine (Invitrogen).

Three different virulent ASFV strains were used for *in vivo* experiments: the heterologous field isolates BA71 and E75 (20) and the Georgia 2007/1 strain, kindly provided by Linda Dixon at The Pirbright Research Institute (UK). Field isolates were grown normally in swine alveolar macrophages and exceptionally in COS-1 cells. Recombinant ASF viruses were all derived from the virulent BA71 strain, grown normally in COS-1 cells and on specific occasions in PAMs. BA71V, the Vero cell-adapted BA71, was grown in Vero cells.

### Animals and animal safety.

*In vivo* experiments were performed in the biosafety level 3 plus (BSL3+) facilities at the Centre de Recerca en Sanitat Animal (IRTA-CReSA; Barcelona, Spain), using 6- to 8-week-old male farm pigs (Landrace × Large White). Animal care and procedures were carried out in accordance with good experimental practices and under the supervision of the Ethical and Animal Welfare Committee of the Universitat Autònoma de Barcelona.

### Experimental design.

Groups of 6 pigs (minimum number per group) were intramuscularly immunized in the neck with 1 ml of the corresponding ASFV dose and virus isolate diluted in PBS. Control pigs received PBS alone. Cell-free extracellular recombinant ASFV was semipurified by centrifugation on a 25% sucrose cushion for *in vivo* immunizations. ASFV challenges were performed with 20 LD_50_ (median lethal doses) of the corresponding field virulent isolates. Cell-free supernatants obtained from ASFV-infected cells were always used for ASFV challenges. Pigs were bled and nasal swabs were taken before and after immunization (4, 7, 14, and 24 days postimmunization [dpi]) or after ASFV challenge (4, 7, 14, and 24 days postchallenge [dpc]). Animals were observed daily according to a welfare schedule to monitor their health status and to record the clinical signs after the infection with ASFV (66). Postmortem examinations were carried out to confirm or discard the presence of ASFV-compatible pathological lesions, although no score was established.

### ASFV titration.

Viremia was quantified in sera as described previously (20, 26, 27). Sera or nasal swabs were collected at different times before and after virus challenge and viremia was quantified using a real-time quantitative PCR (qPCR) method previously described by our laboratory (27). Briefly, the viral genomic DNA was obtained from 200 μl of sera or swab-PBS suspensions using the NucleoSpin blood kit (Macherey-Nagel) and then used as the template to amplify an 85-bp-long fragment from the ASFV serine protein kinase gene (*R298L*). PCR amplifications were performed in duplicates using the corresponding standards for absolute quantification. The results were expressed as log_10_ genome-equivalent copies (GEC) per milliliter of sera or nasal swab, and the detection limit of the technique was 10^3^ GEC/ml. The results of the qPCR showed a slope (*r*) of 0.98 in correlation with the results of the hemadsorbing assay (OIE-validated assay) in serum samples. Alternatively, ASFV was titrated by plaque assay in COS-1 cells as previously described (39). Briefly, COS-1 cell monolayers were infected with serial dilutions of the sample and then covered with 0.7% melting agarose in DMEM plus 2.5% FCS. Five to 7 days later, plates were fixed with 5% formaldehyde and stained with 2% crystal violet for ASFV plaque visualization. ASFV titers were quantified as PFU per milliliter. For recombinant selection, plates were alternatively incubated with 300 μg/ml of either 5-bromo-4-chloro-3-indolyl-β-d-glucuronic acid (X-Gluc; substrate for the β-glucuronidase enzyme) or 5-bromo-4-chloro-3-indolyl-β-d-galactopyranoside (X-Gal; substrate for the β-galactosidase enzyme), and blue-stained plaques were selected from the agar monolayer for further purification.

### Immunological readouts.

ASFV-specific antibodies in pig sera were detected by the OIE-approved ELISA based on soluble extracts from ASFV-infected cells (67). The presence of positive sera was detected using a peroxidase-conjugated anti-pig IgG at a 1/20,000 dilution (Sigma-Aldrich) as a secondary antibody and soluble 3,3′,5,5′-tetramethylbenzidine (TMB) as a specific peroxidase substrate (Sigma-Aldrich). Reactions were stopped with 1 N H_2_SO_4_ (Sigma-Aldrich), and the ELISA plates were read at a wavelength of 450 nm (λ_450_). The results were represented as the average absorbance (optical density [OD] values) of duplicates.

The frequency of ASFV-specific IFN-γ-secreting cells (IFN-γ-SC) in PBMCs was analyzed by an IFN-γ ELISPOT assay using commercial monoclonal antibody tandems (swine IFN-γ; Cytoset) and following a previously reported method (27). Briefly, porcine PBMCs were specifically stimulated for 20 h *in vitro* with 10^6^ 50% hemadsorbing units (HAU_50_)/ml of BA71ΔCD2, using RPMI 1640 and 10 μg/ml of phytohemagglutinin (PHA; Sigma-Aldrich) as negative and positive controls, respectively. The frequency of ASFV-specific IFN-γ-SC per million PBMCs was calculated subtracting the spot counts in the negative-control wells (stimulated with medium alone). Any sample scoring ≥300 spots/million PBMCs received a score of 300 (considered the limit of our assay resolution).

PBMCs labeled with carboxyfluorescein diacetate succinimidyl ester (CFSE; Vybrant CFDA SE Cell Tracer kit; Invitrogen) were alternatively stimulated for 5 days with 10^6^ HAU_50_/ml of BA71 or E75, two heterologous ASFV strains (20). After the stimulation, viable cells were labeled with LIVE/DEAD fixable violet dead cell stain kit (Invitrogen) and surface stained with anti-CD8-Alexa Fluor 647 (clone 76-2-11) and anti-CD4-peridinin chlorophyll protein (PerCP)-Cy5.5 (clone 74-12-4) antibodies (both from BD Pharmingen, NJ), which allowed the identification of the phenotype of the proliferating specific T cells (27, 68).

### Construction of ASFV recombinant virus BA71ΔCD2.

The EP402R upstream (1,052 bp) and downstream (1,020 bp) flanking regions containing the EP152R-EP153R and EP364R genes, respectively, were amplified by PCR using the oligonucleotide pairs J059/J060 and J061/J062 (Table 2), respectively, and BA71 genomic DNA as the template.

**TABLE 2:** Oligonucleotides used for PCR amplification

Oligonucleotide	Sequence (5′→3′)
J059^*a*^	GATGGCTCGAGTTTTTCAGCAAGATCTCGACGTAATAACATTTTACACG
J060^*a*^	CCGTCGAGGGTACCGAGCTCGAATTACATATTGTTTAATTTATCATTATTTACCA
J061^*b*^	TAGAGTCGAGTTTTTTTTTTCAGCTAATATTTCGCTTATTCATGTAGATAG
J062^*b*^	ATTGTAGGAGATCTTCTAGAAAGATCTTCATTCCCAACTTAATCGT

a Upstream flanking region.

b Downstream flanking region.

**FIG 8:**
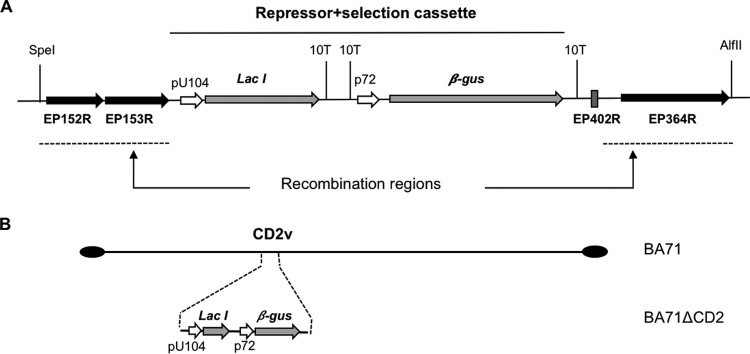
Schematic representation of pLacI.ΔCD2 plasmid and recombinant virus BA71ΔCD2. (A) The pLacI.ΔCD2 plasmid, the LacI repressor gene under the control of the ASFV early/late promoter pU104, and the marker β-glucuronidase gene under the control of the late p72 promoter are flanked by the recombination regions that consist of the EP152R and EP153R genes (upstream) and the EP364R gene and a 36-bp region of the EP402R gene (downstream). (B) BA71ΔCD2 recombinant virus lacks the EP402R gene (encoding the CD2v protein) and instead contains the LacI repressor gene under the control of the ASFV early/late promoter pU104 and the marker β-glucuronidase gene under the control of the p72 promoter.

Amplified fragments were digested with the appropriate restriction enzymes and cloned into the LacI.10T.βGUS.10T plasmid (31) using the Gibson assembly cloning kit (New England BioLabs [NEB]). The final plasmid, pLacI.ΔCD2, contains, between the EP402R upstream and downstream sequences (Fig. 8), the repressor plus selection cassette consisting of the LacI repressor gene under the control of the ASFV early/late promoter pU104 and the marker β-glucuronidase gene under the control of the late p72 promoter.

BA71ΔCD2 (Fig. 8) was generated by homologous recombination between the pLacI.ΔCD2 plasmid and the BA71 ASFV genome as previously described (31), with minor modifications. Briefly, 100,000 COS-1 cells were transfected with 500 ng of pLacI.ΔCD2 plasmid using Lipofectamine (Invitrogen) according to the manufacturer's recommendations. After 3 h, cells were infected with BA71 at a multiplicity of infection (MOI) of 3 until total cytopathic effect was achieved. The cells were harvested and the recombinant viruses were isolated by sequential rounds of plaque purification in the presence of 300 μg/ml of X-Gluc.

BA71ΔTK and BA71ΔTKv220i were obtained using plasmids and procedures previously described to obtain identical recombinants using the Vero cell-adapted BA71 virus (BA71V). Briefly, COS-1 cells were first transfected with the plasmid pINSGUS (31), containing the repressor plus selection cassette between the thymidine kinase (TK) gene upstream and downstream sequences and next infected with BA71 to obtain BA71ΔTK, lacking the TK gene. Once obtained, BA71ΔTK was used to infect fresh COS-1 cells previously transfected with pIND2.pp220 (37), a plasmid that contains a cassette formed by the *lacZ* gene under the control of the strong late promoter p72 and the CP2745L gene (encoding the pp220 protein) under the control of the virus-inducible promoter p72.I. The resulting recombinant virus, BA71ΔTKv220i, was viable only in the presence of the IPTG inducer, confirming the essentiality of the CP2745L gene.

### Sequencing and data analysis.

Viral genomic DNA was extracted from semipurified BA71ΔCD2 preparations using the Nucleospin viral RNA and DNA isolation kit according to the manufacturer's instructions (Macherey-Nagel) and sent to Servei de Genomica from the Universitat Autònoma de Barcelona for Illumina paired-end 2 × 250-bp sequencing according to the manufacturer's instructions (MS-102-2003 MiSeq reagent kit v2; 500 cycles). Read pairs were quality checked and joined using fastqjoin software (69). *De novo* assembly was done using A5 assembler pipeline (70) under the default parameters, and the scaffold_builder tool (71) was used for scaffolding the contigs. The assembled genome was compared with that of the ASFV parental virulent BA71 strain (GenBank accession number KP055815.1) (46) using Artemis Comparison Tool (72), and Gepard software 1.4v was used to calculate dot plots for genome comparisons (73). The BA71 sequence (GenBank accession number KP055815.1) was also used as the reference to perform the mapping of the joint reads with bwa (74), using the mem algorithm. Samtools utilities (75) were used for calculating coverage, for single nucleotide polymorphism (SNP) calling, and to further process files for manual inspection using the Integrative Genomic Viewer (76, 77).
